# Long Live the King!

**Published:** 1910-05-14

**Authors:** 


### Long Live the King!

The King is Dead! Long Live the King!

While the nation mourns the loss it has sustained through the death of Edward the Peacemaker, the beloved Sovereign who during his short reign has endeared himself to his people, it is called upon loyally to acclaim the new ruler and to lose, for a moment, its grief in a general and fervent expression of subdued but sincere congratulation. There is no contradiction in this conjunction of condolence and well-wishing. The old order passes and yields place to the new: the dead King lives only in the memory of his subjects, the living Sovereign is the head and chief of his nation. So, reverently and solemnly, the closing sentences of the King's funeral service will voice the general sentiment in the old formula: "Inasmuch as it hath pleased Almighty God to take from the troubles of this life the most high, mighty, and excellent monarch Edward . . . let us therefore beseech Almighty God to bless with long life, honour, and all worldly blessings the most high, mighty, and excellent monarch George." With this prayer every British subject at this moment is in unison, and the Queen-Mother well knows that her confidence in the people, to whose care, loyalty, and devotion she has commended her son, will not be in vain nor misplaced. Assuredly his will be the love of his nation and the devotion of those who owe him allegiance, for to them all the new Sovereign is already a familiar figure who has shown himself fully conscious of the responsibilities of kingship.

King George succeeds to his glorious heritage at a time when men's hearts are profoundly stirred and when movements for political and social alteration have shaken the whole of his kingdom. As Sovereign, standing apart and impartial, it is usually supposed that the King has nothing to do with these things, that they do not affect him, and that he has no voice in their consideration, no say in any decision on subjects which so profoundly touch the national welfare as do the great problems of the day. To admit that were to deny to kings the privilege of being human, of feeling, sympathising, and acting with their people. No one who has followed the new king's career before he came to the Throne can for a moment doubt that, like his father, he will make his voice heard in the councils of the nation and his influence felt in the furthest corners of this vast Empire. He has already shown that he possesses the faculty of going to the root of a question, of trying to solve problems logically, and of finding the right words aptly to express the needs of the moment. His public utterances have proved that he has thought deeply and conscientiously over matters which are of vital importance to the nation. No king, we venture to assert, ever ascended the Throne of England with so intimate a knowledge of his dominions, of the races and peoples subject to him, of their relations to the Mother-country, and of their ideals, hopes, and aspirations. He has trod British soil nearly in every part of the world where the Union Jack flies, and acquainted himself, by actual intercourse with the citizens in every Dominion and Colony, with their interests and aims. He has realised, as no sovereign before him perhaps has realised, the inherent truth in Milton's aphorism that "there is a mutual bond of amity and brotherhood between man and man over all the world; neither is it the English sea that can sever us from that duty and relation, and yet a straiter bond there is between fellow-subjects, neighbours, and friends." He ascends the Throne at a time when there is peace throughout his dominions, when his kingdom is consolidated as at no period of its history it has ever been, and it is unfortunate that one of his first duties means the unavoidable but regrettable cancellation of a mission that, we feel sure, would have cemented yet closer the bonds which bind the newest Dominion overseas to this country. It is sincerely to be hoped that some means may yet be devised to give his son, Prince Edward, the opportunity of undertaking that mission and acting in his stead when the time comes for the final culmination of the generous policy ratified by King Edward VII.

As a publicist the King is already well known. As a hospital president and governor he is deservedly popular and appreciated, for his custom has always been to take his office seriously and to fulfil the duties thereunto appertaining not by deputy, but by giving his personal service to the affairs of the charities privileged to possess him as their chief authority. As President of the King's Fund and of the League of Mercy, his work on behalf of the hospitals has been singularly beneficial. Much of the success of the Fund and League is due to his able direction of their activities and to his inspiriting encouragement of all who have devoted themselves to their development and expansion. In him the hospitals will ever have a staunch friend, an earnest worker, and a liberal supporter, though it is unquestionable that, like his lamented father, he will not suffer his name to be connected with institutions that are not in every respect worthy of the high honour of the Royal patronage.

In all matters concerning charity, as in all matters affecting the weal of his subjects, he will be ably supported by Queen Mary. She, too, has proved herself a thoughtful and warm-hearted friend of hospitals; she has given up her time to their inspection and has become the patron of many institutions. By her personal visitation she has familiarised herself, as Queen Alexandra has done, with the conditions under which the poor are cared for in our charitable institutions, and has cheered and comforted many sufferers by her kindly attention and generous sympathy. To the nation at large she is known as the constant helper and unwearied coadjutor of her husband. She accompanied him on his travels, and it is no exaggeration to say that the Queen is as well known and as beloved in the dominions overseas as she is in these islands.

To-day, when Their Majesties the King and Queen are remembered everywhere in the prayers of their people, no single subject will omit to wish them prosperity, happiness and all worldly blessings, honour, and length of days. The King has a glorious example to guide him in the memory of what his beloved father accomplished for the Empire, for the nation, and for humanity. That he may win, as King Edward won, the right to be acclaimed the father of his people, guarding their weal, cherishing their welfare, and working for their interests, is the best wish that every loyal subject can tender him in this day of his sorrow and his succession.

